# A new method based on diffusive gradients in thin films for *in situ* monitoring microcystin-LR in waters

**DOI:** 10.1038/s41598-019-53835-6

**Published:** 2019-11-26

**Authors:** Lei Yao, Alan D. Steinman, Xiang Wan, Xiubo Shu, Liqiang Xie

**Affiliations:** 1 State Key Laboratory of Lake Science and Environment, Nanjing Institute of Geography and Limnology, Chinese Academy of Sciences, Nanjing, 210008 China; 2 College of Resources and Environment, University of Chinese Academy of Sciences, Beijing, 100049 China; 3 Annis Water Resources Institute, Grand Valley State University, 740 West Shoreline Drive, Muskegon, MI 49441 USA; 4 College of Resources and Environment Engineering, Guizhou University, Guiyang, 550025 China

**Keywords:** Environmental monitoring, Environmental monitoring, Environmental impact, Environmental impact

## Abstract

The passive sampling method of diffusive gradients in thin-films (DGT) was developed to provide a quantitative and time-integrated measurement of microcystin-LR (MC-LR) in waters. The DGT method in this study used HLB (hydrophilic-lipophilic-balanced) material as a binding agent, and methanol as an eluent. The diffusion coefficient of MC-LR was 5.01 × 10^−6^ cm^2^ s^−1^ at 25 °C in 0.45 mm thick diffusion layer. This DGT method had a binding capacity of 4.24 μg per binding gel disk (3.14 cm^2^), ensuring sufficient capacity to measure MC-LR in most water matrices. The detection limit of HLB DGT was 0.48 ng L^−1^. DGT coupled to analysis by HPLC appears to be an accurate method for MC-LR monitoring. Comparison of DGT measurements for MC-LR in water and a conventional active sampling method showed little difference. This study demonstrates that HLB-based DGT is a useful tool for *in situ* monitoring of MC-LR in fresh waters.

## Introduction

As cyanobacteria (blue-green algae) grow in eutrophic waters, various toxins can be excreted from live or dead cells^1,2^. One category of toxins is microcystins (MCs), a hepatotoxin containing cyclicheptapeptides^3^, which can be released from several species of cyanobacteria. Microcystin-LR (MC-LR) is the one of most harmful among the 100 or more microcystin congeners^1^, and is also the most frequently reported MC^4^. The maximum allowable concentration (MAC) for MC-LR in drinking water was established as 1 μg L^−1^ ^5^. Reported concentrations of MC-LR in Taihu Lake (China) range from 0.05–3.38 μg L^−1^ ^6–9^. MC-LR has wide-ranging negative impacts on organisms, including humans^3,10–12^. A Tolerable Daily Intake (TDI) value of 0.04 μg kg^−1^ MC-LRof body weight per day was proposed as a provisional guideline by the WHO (World Health Organization)^13^. Therefore, precise monitoring is essential to understand MC fate and behavior, and to provide data to assess possible risks to human health and ecosystems.

Inactive sampling methods, where water or sediment samples are collected on site and transferred to the lab for detection and measurement of concentration, is a common procedure for many organic chemicals, such as BPs (bisphenolA)^14^, PCBs (polychlorinated biphenyls)^15^, and MCs^4^. An active sampling approach provides a concentration at a discrete time, but also usually involves a large investment of time and cost. In contrast, passive sampling methods have been developed in recent decades, and partly through *in situ* deployment^16^ (Gong *et al*., 2018), can overcome active sampling shortcomings, such as less pretreatment of samples, higher resolution than active sampling methods, and lower requirements of experimental skills. Passive sampling methods are complementary to direct sampling of water and biological samples. Dominant passive sampling approaches include the Polar Organic Chemical Integrative Sampler (POCIS)^17^, Chemcatcher^18^, Solid Phase Adsorption Toxin Tracking (SPATT)^19^, and Diffusive Gradients in Thin films (DGT)^20^. Both POCIS and SPATT have been deployed successfully to detect or monitor microcystins^21–23^, providing a robust way to monitor microcystin concentrations. But according to a previous report^24^, under some conditions, POCIS may result in inaccurate estimations of organic compound concentrations. SPATT also presents some limitations, such as a lack of calibration (no optimal deployment time), thereby limiting the more widespread adoption of this technology for monitoringof pollutants^25^. The DGT technology was developed by Davison and Zhang^26^ and initially was used to detect trace metals in natural fresh water systems^20^.

Recently, Chen *et al*.^27,28^ and Zheng *et al*.^29^ have successfully used this technology to measure antibiotics and BPAs in waters. Chen *et al*.^30^ used HLB (hydrophilic-lipophilic-balanced, OASIS) as the binding layer in DGT to measure HPCPS (house-hold and personal care products) in waters. Zou *et al*.^31^ also used HLB in DGT to detect organo-phosphorus flame retardants. The HLB material is a well-known and effective adsorbing material for several organics, including MCs^32^.

In the present study, we prepared the binding gel with HLB incorporated into agarose gel. In assessing the performance characteristics of the new DGT device for detecting extracellular MC-LR, we studied the binding kinetics, elution efficiency using four different eluents, capacity of the binding gels, and the possible influence of lake water pH and ionic strength. The DGT devices employing HLB gels were tested in natural waters, and the resulting concentrations were compared with conventional active sampling.

## Results and Discussion

### Adsorption by experimental devices

All materials used in the experiment should be non-adsorptive and non-reactive with respect to MC-LR. Hence, the diffusive gels, DGT models, filter membranes, and polypropylene pipes (as an efferent vessel for the extraction process) were assessed for their possible adsorption of MC-LR. The results of the adsorption tests indicated that there was a limited adsorption ratio by the DGT models (0.26%), polypropylene pipes (0.22%), diffusive gels (0.43%), and PTFE filter membranes (0.23%). These results suggest that the four materials can be used without worry of adsorption by materials used in the experiments.

### Sorption kinetics of MC-LR

**Figure 1:**
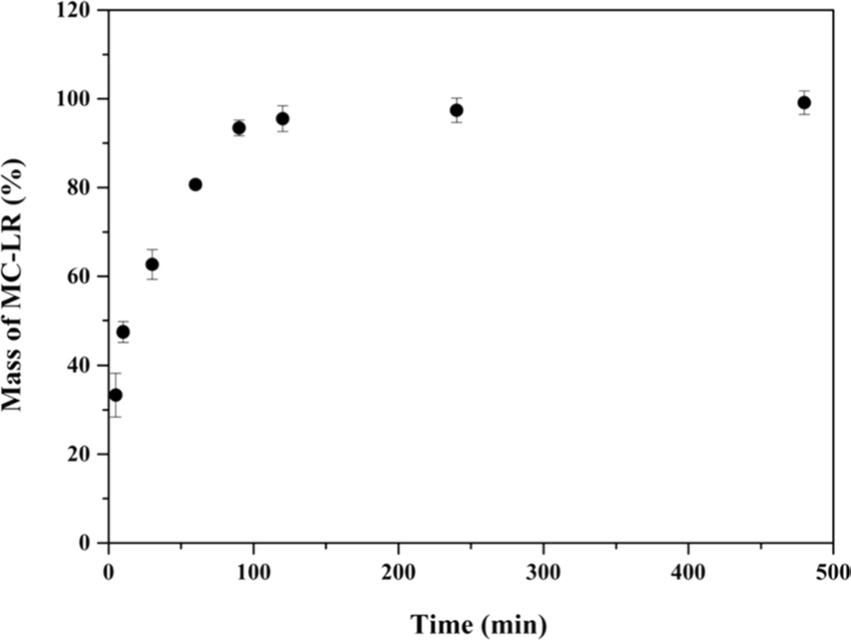
The mass of MC-LR adsorbed by binding gel in 20 mL solutions containing 200 μg L^−1^ MC-LR and 0.01 M NaCl. The Y axis refers to the ratio of mass of MC-LR adsorbed by the HLB gel divided by the mass contained in the initial solutions. Error bars are calculated from the standard deviation of the replicates (n = 3).

The uptake of MC-LR by the binding gel increased quickly during the first 60 min, and then reached a plateau by approximately 90 min, when more than 95% of the MC-LR had been adsorbed (Fig. 1). The average binding rate calculated from the DGT unitwas0.59 ng cm^−2^ min^−1^ when they were deployed in 200 μg L^−1^ of MC-LR at 25 °C. The average binding rate of the binding gel during the first 30 min was 3.09 ng cm^−2^ min^−1^, which was sufficient to ensure uptake by the DGT. Similar results were reported by Zheng *et al*.^29^ using activated charcoal binding gels for measuring BPs. The adsorption rate discrepancy between DGT device and binding gel suggests that MC-LR binds onto the gels in DGT with sufficient speed to ensure that the concentration of MC-LR at the diffusive gel/binding gel interface is almost zero, validating the use of Eq. (1).

### Elution efficiencies of MC-LR

**Figure 2:**
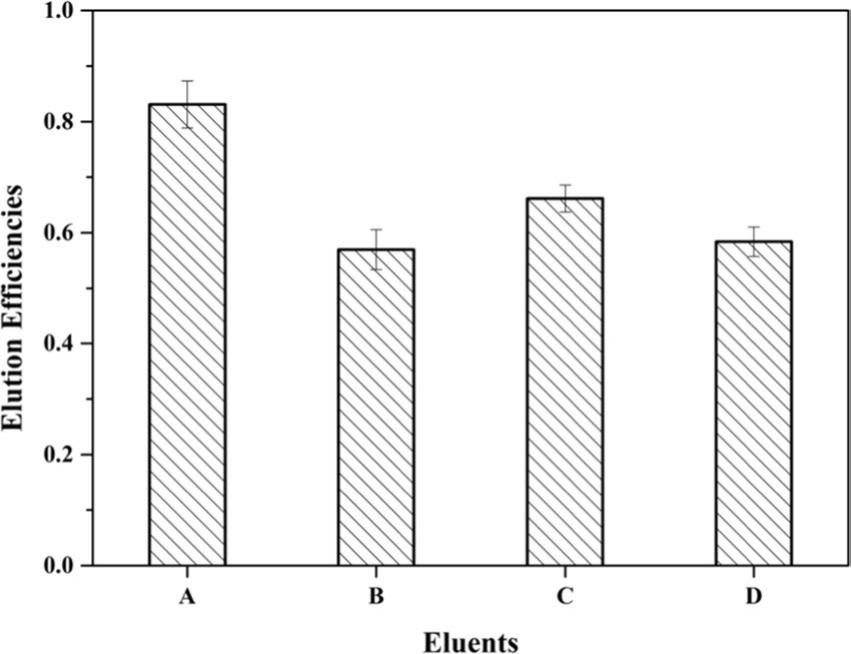
Elution Efficiencies (%) of MC-LR from Binding Gels Eluted by Different Eluents (A:10 mL of methanol; B:7 mL of methanol + 3 mL of 1 M NaOH; C:9 mL of methanol + 1 mL of 1 M NaOH; D:7 mL of methanol + 3 mL of 1 M HCl, Error bars are calculated from the standard deviation of the replicates (n = 3).

Stable and high elution efficiency of MC-LR from HLB gels was needed to calculate *C*_DGT_. In the present study, four kinds of eluents were tested to identify the most suitable one. Eluent A, consisting of 10 mL of methanol only, had the highest elution efficiency (83.09 ± 4.2%) (Fig. 2). The other three eluents had lower efficiencies, ranging from 56.9 ± 3.58% to 66.1 ± 2.42%.

### Diffusion coefficient

The effective diffusion coefficient of MC-LR was determined by the DGT time-series deployment method. This method is more widely used for the calculation of the diffusion coefficient than the diffusion cell method because it more closely reflects natural conditions^33^. The accumulated mass of MC-LR was linearly correlated with the deployment time of DGT units. The slope fitting equation was used to calculate the effective diffusion coefficient according to Eq. 3. The diffusion coefficient of MC-LR was 5.00 × 10^−6^ cm^2^ s^−1^, at 25 °C in the 0.45 mm thickness diffusion layer. This was similar to the diffusion coefficient of BPA (slightly higher than BPB, lower than BPF) measured using an agarose diffusive gel reported by Zheng *et al*.^29^. A prior study showed the diffusive coefficient of several HPCPs ranged from 3.36 × 10^−6^–7.30 × 10^−6^ cm^2^ s^−1^ at 25 °C using the agarose diffusive gel^30^.

### DGT blanks and detection limits

DGT blank concentrations were evaluated by measuring the mass of MC-LR, using HPLC, in HLB gels retrieved from DGT units that were deployed in 0.01 M NaCl solution for 24 h. No MC-LR was detected. A method detection limit (MDL) for HLB DGT of 0.48 ng L^−1^ was calculated assuming a deployment time of 96 h at 25 °C with a 0.45 mm thick diffusive gel. The reported concentrations of MC-LR were 0.05–2 μg L^−1^ in Taihu Lake^6^. Given the much lower values of the MDL than the reported natural concentrations, DGT coupled to analysis by HPLC appears to have adequate sensitivity for water quality monitoring. A longer deployment time could be used to enhance the adsorbed mass and lower the MDL proportionately if the concentration of MC-LR was lower than the MDL^34^.

### Effects of pH and ionic strength

**Figure 3:**
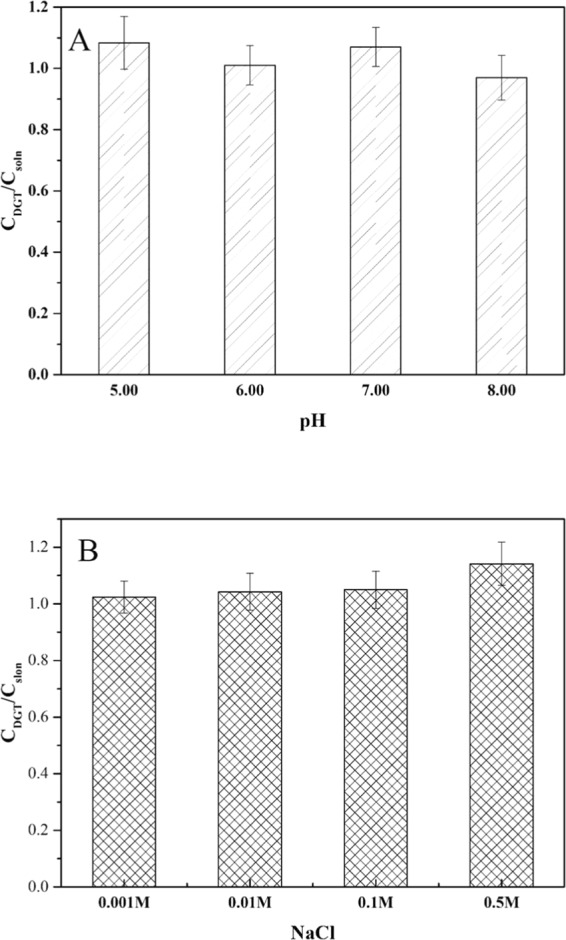
Effect of pH (**A**) and ionic strength (**B**) on the ratio of DGT-measured concentrations (*C*_DGT_) to their concentrations in bulk solutions (*C*_soln_) (there were no statistically significant differences among the pH and IS treatment levels in each panel), error bars are calculated from the standard deviation of the replicates (n = 3).

According to Kim *et al*.^35^, pH has a considerable impact on MC-LR adsorption when the compound contains many carboxyl, phosphonate groups, and amine groups, but has little effect on neutral compounds^36^. The performance of the HLB DGT for measuring MC-LR in solutions with various pH and ionic strength (IS) conditions is shown in Fig. 3. A pH range from 5 to 8 had no obvious effect on the measurement of MC-LR by DGT. This suggests that DGT performance is generally independent of solution pH within this range and hence, DGT can be directly used for MC-LR measurement in field conditions that have a pH of 5–8.

There was no observed effect of the different IS on MC-LR (Fig. 3) adsorption experiment. IS could potentially affect the adsorption process of several organics, such as sulfachloropyridazine, tylosin, and oxytetracycline^37^, but has no discernible effect on materials without significantly ionized forms^38,39^. The result that IS has little effect on adsorption is consistent with previousstudies^36^. Measurement of BPs using DGT with activated charcoal binding gel also was unaffected at an IS of 0.001–0.05 M^29^. While the DGT with XAD18 (styrene divinylbenzene copolymer) binding gel to detect the antibiotic sulfamethoxazole was unaffected at IS from 0.001–0.01 M, it was affected at a concentration of 0.05 M^28^. These findings indicate that the DGT unit with HLB binding gel for measuring MC-LR is suitable in freshwater, and some work has been done by Xie *et al*.^40^ in seawater, still further work is needed on the effect of IS before DGT is applied in different matrices.

### Capacity for DGT response

**Figure 4:**
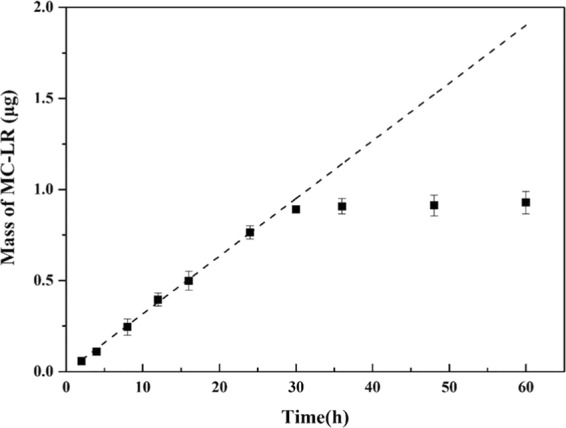
Measured masses of MC-LR adsorbed onto the HLB gels in DGT units deployed in well-stirred solutions for different times with an MC-LR concentration = 200 μg L^−1^. The dashed line is predicted from the known solution concentrations using Eq. (1), error bars are calculated from the standard deviation of the replicates (n = 3).

To ensure an accurate measurement by DGT when they are deployed at high concentrations or over the long-term, the binding phase must have sufficient capacity to avoid saturation. The mass of MC-LR adsorbed on the HLB gel within the DGT units increased linearly from 0–24 h and was close to the theoretical 1:1 line (Fig. 4). The adsorption capacity of DGT with HLB binding gel for MC-LR was 1.35 µg cm^−2^. Based on this capacity, if deployed for 2 weeks, the maximum concentration which could be retained would be 10 µg L^−1^; if deployed for 1 month, the maximum concentration would be 4.6 µg L^−1^. However, much shorter deployment times would likely be used to avoid the possibility of biofouling or other disturbances in the environment. In most situations, MC-LR concentrations in polluted waters would be less than 10 µg L^−1^, so the capacity of DGT devices equipped with HLB binding gel is therefore more than adequate for monitoring MC-LR in most natural environments.

### Testing in natural waters

In this study, the thickness of DBL (Diffusive Binding Layer, *δ*) is not negligible, and was calculated based on Warnken’s^41^ method (Eq. 1 and Eq. 2). After 7 days, the R^2^ of 1/M (μg^−1^) versus Δ*g* (mm) plots and the DBL thickness (*δ*) based on the field data were calculated. The calculated DBL thickness (*δ*) was 0.21 mm.

1 $$\partial =\frac{b}{m}(\frac{{D}_{w}}{{D}_{gel}})$$

2 $${C}_{DGT}=(1/m{D}_{gel}At)$$

**Figure 5:**
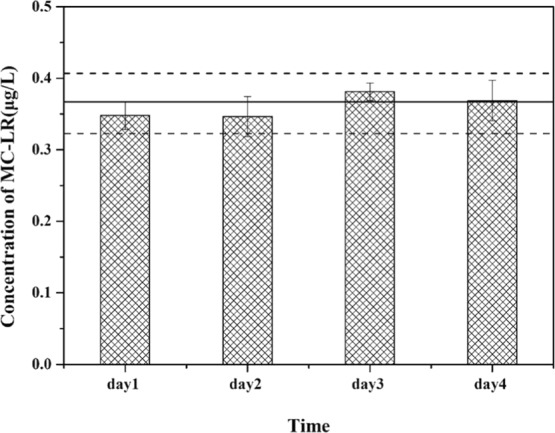
The concentrations of MC-LR during a 4 day sampling period. The solid line is the time-averaged concentrations of MC-LR detected by DGT. The dotted lines represent the maximum and minimum concentrations of MC-LR detected by DGT. The bar graph represents the concentrations of MC-LR detected by the active sampling method, error bars are calculated from the standard deviation of the replicates (n = 3).

The DGT units were deployed in Taihu Lake (Meiliang Bay). The concentrations of MC-LR detected by active sampling were 0.276–0.311 μg L^−1^ over the four days, while the result of the passive DGT method was 0.294 μg L^−1^. This MC-LR concentration is similar to those reported for other systems, including Meiliang Bay in Taihu Lake^4^ and Oneida Lake^42^, which were both measured with the active sampling method. The MC-LR concentrations obtained through the passive sample method generally fell within the minimum and maximum concentrations detected by the active method (Fig. 5), suggesting that the DGT with HLB binding gel is an appropriate tool for detecting MC-LR in natural waters.

## Methods

### Materials and reagents

The DGT detection result, *C*_DGT_, provides a dynamic concentration of organic materials in the solution using Fick’s first law of diffusion^26^:

3 $${C}_{DGT}=\frac{M(\Delta g+\delta )}{DAt}$$

Where *M* (μg) represents the measured mass of a target material adsorbed on the binding gel, *∆g* (mm) is the thickness of the diffusion layer, *δ* (mm) is the DBL thickness, *D* (μg cm^−2^ s^−1^) is the diffusion coefficient of the target material, *A* (cm^2^) represents the area of the DGT device exposed to the aquatic matrix, and *t* (s) represents the time that the DGT device was deployed in the aquatic matrix.

The DGT moldings of ABS (acetonitrile-butadiene-styrene) were obtained from DGT Research Ltd., United Kingdom. The self-made holders (cylindrical, 2 L, 30 cm high) for deployment of DGT devices were made of polytetrafluoroethylene (PTFE). Stock solutions (in water) of MC-LR (Taiwan Algal Science Inc.) were prepared at 50 mg L^−1^ and stored at 4 °C. Methanol (HPLC grade) was purchased from Merck China. HLB was purchased from Waters Oasis, USA. The PTFE filter membrane (diameter 25 mm, pore size 0.45 μm, thickness 150 μm) was purchased from Shanghai Anpel Scientific Instrument Co. The polypropylene (PP) pipes (Corning, USA) were used to extract the MC-LR from binding gel into the methanol. Ultrapure water (Thermo Scientific) was used during the whole experiment.

### Chemical analysis

Quantification of MCs was performed on an Agilent 1000 series high performance liquid chromatography (HPLC) system with a DAD detector (Agilent, Palo Alto, CA, USA) according to the method of Su *et al*. (2015). Briefly, MCs were extracted by using HLB cartridges and measured by HPLC. The HLB cartridges were previously activated. The samples (filtered before) were then applied at a flow rate of 1 mL min^−1^, with a further washing step with 5% (v/v) methanol, and a final elution with methanol was performed. A volume of 15 mL was used for the washing step and 10 mL for the elution step described above. Finally, the eluent was dried under N_2_ gas at 40 °C prior to reconstitution in 1.0 mL of methanol. A 500 μL sample was prepared for HPLC analysis. The extracted solution of DGT units was injected into the HPLC without pre-concentration.

### Evaluation of possible adsorption

DGT moldings, diffusive gels, PTFE filter membranes, and PP pipes were soaked in 200 mL of 200 μg L^−1^ MC-LR solutions and then placed on a shaking table for 24 hours at 200 cycles per minute at room temperature. The concentrations of MC-LR in the solutions before and after exposure were measured, and the difference calculated to obtain the mass adsorbed.

### Diffusive and binding gel preparation

The diffusive gel was made according to Zheng *et al*.^29^. Briefly, 0.15 g agarose was dissolved in 10 mL water and heated to 100 °C to create a 1.5% agarose solution. The hot solution was injected into pre-warmed gel-casting molds, which includes two sheets of glass separated by 0.45 mm spacers (made of polyethyleneterephthalate). The solution was left to cool to room temperature. The gels were stored in 0.01 M NaCl solution, and were cut to the desired shape before use; the rounded cutting knife was made of Hadfield Steel. The binding gel was made by mixing 400 mg of HLB in 10 mL of 1.5% warm agarose solution. The mixture was injected into preheated molds (two glass sheets separated by 0.45 mm thick spacers), then left to cool to room temperature. The binding gels were stored in ultrapure water and cut into disks before use.

### Binding kinetics and elution efficiency

The HLB binding gel disks were immersed and shaken in 20 mL of 200 μg L^−1^ MC-LR (0.01 M NaCl, pH = 7.0)for time intervals of 5 min, 10 min, 20 min, 30 min, 60 min, 90 min, 120 min, 240 min, and 480 min. Four kinds of eluents were selected: A: 10 mL of methanol; B: 7 mL of methanol + 3 mL of 1 M NaOH; C: 9 mL of methanol + 1 mL of 1 M NaOH; and D: 7 mL of methanol + 3 mL of 1 M HCl. The binding gels loaded with MC-LR were extracted for 24 h at 25 °C. The eluents were injected directly into the HPLC to obtain the elution efficiency.

### Diffusion coefficient

The diffusion coefficient of MC-LR in the agarose gel was estimated during a DGT time-series deployment experiment. The HLB DGTs were deployed into 2 L mixed solutions containing 0.01 M NaCl and 200 μg L^−1^ MC-LR (pH = 7) at 25 °C. The DGT devices were deployed and retrieved at several time intervals ranging from 4 h to 24 h. The binding gels were rinsed with ultrapure water, then eluted and detected by the methods described above. The mass of MC-LR in the binding gel was calculated by Eq. (4):

4 $$M=\frac{{C}_{e}({V}_{g}+{V}_{e})}{{f}_{e}}$$

Where *C*_*e*_ is the MC-LR concentration in the eluted solution, *V*_*g*_ and *V*_*e*_ are Volumes of the gel and eluent, respectively, and *f*_*e*_ is the elution efficiency. The effective diffusion coefficient (*D*, cm^2^ s^−1^; Eq. (5)) was computed from the slope of the linear regression for M, from Eq. (4) as a function of the thickness of the diffusive layer (*∆g*, 0.60 mm), the area of the diffusive window (*A*, 3.14 cm^2^), and the concentration of the solution (*C*_*soln*_, ng mL^−1^).

5 $$D=\frac{slope\cdot \varDelta g}{{C}_{sol}\cdot A\cdot 60}$$

The coefficient measured was specific to 25 °C; however, the effective diffusion coefficient at other temperatures can be corrected using Zhang’s method^20^.

### Effects of pH and ionic strength, capacity

The effects of pH and ionic strength (IS) on the measurement of MC-LR by DGT methods were examined by deploying the DGT units in several 2 L (self-made, cylindrical), well-stirred (magnetic stirring apparatus), and mixed solutions containing 200 μg L^−1^ of MC-LR at 25 °C for 24 h. To evaluate the effect of pH, DGT devices were immersed in 2 L of well-stirred solutions (0.01 M NaCl) with pH of 5, 6, 7, or 8. To evaluate the effect of IS, DGT devices were immersed in 2 L of well-stirred solutions (pH = 7) with IS of 0.001 M, 0.01 M, 0.1 M, or 0.5 M using NaCl.

To measure the capacity for accumulating MC-LR by HLB gels incorporated in DGT, the assembled DGT units were deployed in 2 L well-stirred solutions containing 200 μg L^−1^ of MC-LR (pH = 7, IS = 0.01 M) using the time-series DGT accumulation experiments. The deployment times were 2 h, 4 h, 8 h, 12 h, 16 h, 24 h, 30 h, 36 h, 48 h, and 60 h.

### DGT blanks and method detection limits

To determine the blank of DGT devices for detecting MC-LR, six replicates of the blank DGT units were deployed into 2 L of 0.01 M NaCl solution for 24 h. The concentrations of MC-LR in the blank gels were eluted using methanol (see Results and Discussion for the reason in choosing this eluent). The DGT concentrations were calculated by Eq. (1) using the following parameters: 24 h deployment time, 0.60 mm thickness of the diffusion layer and the DBL thickness, 3.14 cm^2^ exposure area, and the diffusion coefficient for MC-LR at 25 °C.

### Application in natural waters

The DGT units were deployed at Meiliang Bay to calculate the DBL. The pH, temperature and conductivity of water were measured *in situ* every day. Water samples were collected and tested every day. Three sets of DGT samplers with different diffusive layer thicknesses (Δ*g* = 0.45 mm, 0.80 mm, 1.00 mm) with filter (0.15 mm) were deployed for 7 days to determine the DBL according to Warnken’s^41^ method.

The HLB DGT devices were deployed in natural freshwater to evaluate the performance. Four DGT units were assembled leaving the exposure windows outward, then they were placed in Meiliang Bay within Taihu Lake (Jiangsu Province) for 4 days. Meiliang Bay is representative of a eutrophic zone, and is located in the northeast portion of Taihu Lake. The MC concentrations in Meiliang Bay have been detected monthly since 2007. The water temperature was measured every 4 hours. The grab samples of water (1 L) were collected at 10:00 am for 4 consecutive days.

The water samples were transferred to the laboratory within 30 minutes and the concentration of MC-LR was measured following Su *et al*.^7^ as described in section “Chemical Analysis”. The HLB gels with MC-LR were immersed in 10 mL methanol for 24 h. The final eluents from DGT measurements were filtered through a 0.45 μm membrane (aqueous phase) and transferred to 2 mL amber HPLC sample vials for instrument analysis. The accuracy and quality of the analysis was controlled by inserting standard samples (MC-LR 10 μg L^−1^, in methanol) every 10 test samples.

## Conclusion

A novel DGT device using HLB resin has been developed for detection of MC-LR in freshwater. The HLB gel was proved with high capacity and rapid binding kinetics for MC-LR. The DGT units with HLB binding gel was not affected by pH in the range of 5–8 and ionic strength (0.001 M-0.5 M) values. In the field trial, the MC-LR was detected by the HLB-DGT devices and the results showed agreement with the active sampling method. The analysis results indicated that the HLB-DGT probe coupled with HPLC had the benefits of preconcentration and high sensitivity. This novel method was confirmed to be a potentially valuable and powerful tool for measuring trace MC-LR in freshwater and can extend to other environmental matrix.
